# An Acceptance Test for Assistive Robots

**DOI:** 10.3390/s20143912

**Published:** 2020-07-14

**Authors:** Francisco Martín Rico, Francisco J. Rodríguez-Lera, Jonatan Ginés Clavero, Ángel Manuel Guerrero-Higueras, Vicente Matellán Olivera

**Affiliations:** 1 Intelligent Robotics Lab, Rey Juan Carlos University, 28943 Madrid, Spain; jonatan.gines@urjc.es; 2 Group of Robotics, University of León, 24006 León, Spain; fjrodl@unileon.es (F.J.R.-L.); am.guerrero@unileon.es (Á.M.G.-H.); vicente.matellan@unileon.es (V.M.O.)

**Keywords:** assistive robots, acceptance test, dementia

## Abstract

Socially assistive robots have been used in the care of elderly or dependent people, particularly with patients suffering from neurological diseases, like autism and dementia. There are some proposals, but there are no standardized mechanisms for assessing a particular robot’s suitability for specific therapy. This paper reports the evaluation of an acceptance test for assistive robots applied to people with dementia. The proposed test focuses on evaluating the suitability of a robot during therapy sessions. The test measures the rejection of the robot by the patient based on observational data. This test would recommend what kind of robot and what functionalities can be used in therapy. The novelty of this approach is the formalization of a specific validation process that only considers the reaction of the person to whom the robot is applied, and may be used more effectively than existing tests, which may not be adequate for evaluating assistance robots. The test’s feasibility was tested by applying it to a set of dementia patients in a specialized care facility.

## 1. Introduction

The European Union’s (EU’s) population will continue to grow older (EU’s population projected up to 2100. Eurostat https://europa.eu/!RV89yT). The share of working-age people in the EU’s total population is projected to decrease from 65% in 2018 to 55% in 2100. In contrast, the share of those aged 65 years or above in the EU’s total population is projected to increase by 11 percentage points, from 20% at the start of 2018 to 31% by 2100. The share of those aged 80 years or above is calculated to almost triple in the same period from 6% to 15%. This will result in growth in the demand for care for the elderly and the lack of caregivers.

**Figure 1:**
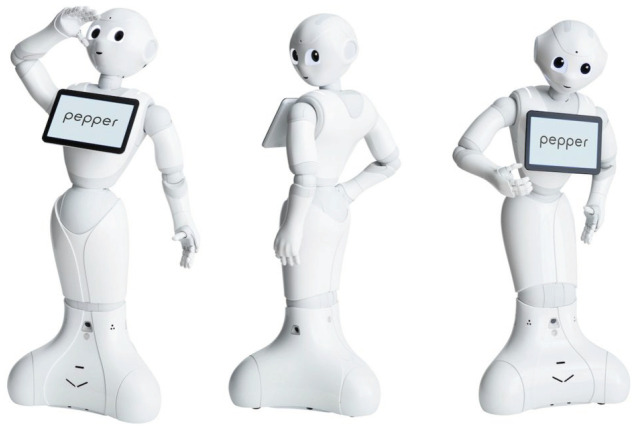
The social robot used in this work.

The idea of using robots in elderly care was launched some decades ago to cope with this problem. Assistive robots for the elderly are usually grouped into rehabilitation robots and socially assistive robots. These robots can either be companion-type robots or service-type robots [1]. This work is focused on the later, and they will be referred to as “assistive robotics” in the rest of the paper in that sense. This test focuses on humanoid robots, and we have used Pepper (https://www.softbankrobotics.com/emea/en/pepper (Figure 1)) in the experiments, a humanoid robot that is explicitly designed to be pleasant to interact with. This robot is widely used in social robotics and it is available worldwide at an affordable price, given its characteristics. The test can be applied to any robot of similar characteristics.

Although there had been many proposals of socially assistive robots for elderly care, their acceptance by the users remains controversial [2]. The morphology and appearance of the robot are known to have a high impact on its acceptance. Many factors influence the acceptability of robot assistance by a patient. In addition to their appearance, the kinematic precision reliability of assistive robots is a crucial factor [3,4,5]. Other relevant factors are size, voice, or even accent [6].

Masahiro Mori contributed in 1970 the disputed “Uncanny Valley Theory” [7]: “I have noticed that, in climbing toward the goal of making robots appear like a human, our affinity for them increases until we come to a valley [...], which I call the uncanny valley”. Although this paper does not deal with the anthropomorphic aspect of robots, it draws from the premise that robot movements have a strong influence on the users’ perception of the robot.

**Figure 2:**
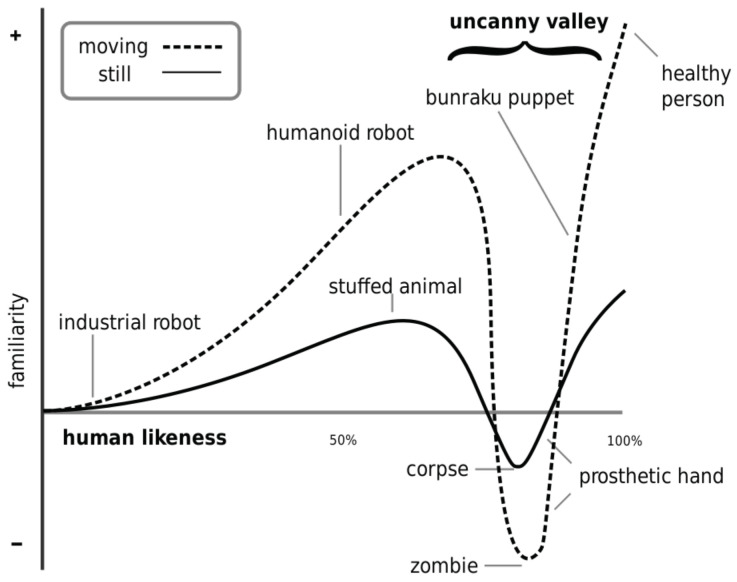
Uncanny Valley Theory.

In this sense, Mori’s theory defined a function of acceptance of a robot depending on the degree to which a robot resembles a human. This function should grow as the resemblance increases. Still, when the robot approaches the human form, the function abruptly takes negative values that indicate a human-to-robot aversion (Figure 2). It is interesting to note that this function takes less extreme values if the robot is stopped. It is then justified that acceptance tests must measure the robot both stopped (or off) and moving.

There have been several other proposals of acceptance tests for robots being used for providing care to elderly users [8]; some are analyzed in more detail in the following section. Franz Werner’s recent review [9] shows that most of the acceptance tests are based on semi-structured interviews and ad-hoc questionnaires. Still, in our opinion, this approach usually takes too long, it is suitable mainly for research analysis, not for robots’ real use.

To provide a test that is more suitable for the use of robots in real scenarios, the work that is described in this paper tries to figure out whether it is possible to establish a non-interview-based method to evaluate the robotic system as social assistants for the elderly, and if this evaluation can be obtained with reduced interaction with the therapist.

The starting hypothesis is that only a few factors have a real influence on the evaluation: the scenario, the patient, and the robot. Because the first two are usually fixed, our proposal is an acceptance test for the robot.

The rest of the paper is organized, as follows. The next section reviews the evolution of acceptance tests for robots used with elderly users. The third section describes the proposed acceptance test, and the following section presents the results of applying it to a group of elderly people with dementia. The fifth section discusses these results, and finally, the conclusions about this research are presented in the last section.

## 2. Related Work

The way to assess people’s attitudes towards robots has usually been made by closed interviews based on questionnaires. One of the most popular in the Human-Robot-Interaction field is NARS (Negative Attitudes towards Robots Scale) [10]. It is based on a questionnaire (Table 1) whose answers define the person’s attitude towards robots. This questionnaire is general, and it is made up of the following items:

In the NARS [11] test, the subject begins by fulfilling the questionnaire. Next, the robot enters the room, stands near the person, and the person has to start to interact with it, both by touching it and through dialogue. The experiment is recorded with two cameras, and the result of the NARS is checked by observing the person’s attitude to the robot. This study concludes that the person’s attitude towards the robot does not always coincide with the NARS scale when interacting with it.

Along the same lines, [12] warns about the difference between attitude tests towards the robot, and expectations about the ideal robot for certain tasks. Indeed, the tests must be adapted to the activity that a robot must carry out. The results of NARS do not always agree with the evaluation in a specific activity. This same opinion is corroborated in [13], where the authors also provide a series of guidelines and factors that influence making this acceptance effective in the long term for a social robot.

The ASOR-5 scale (Attitudinal Stance Towards Social Robots) [14] is used to a minimal extent in social robotics contexts. Although it does not provide enough information to create a complete questionnaire, it has inspired works such as [13], which studies the impact of a robot in residential environments. This study compares the perception of the robot between people who know that the robot is teleoperated, and those who do not. The result indicates that there is no substantial difference between both populations.

The RoSAS (Robotic Social Attribute Scale) [15] is another widely used scale in social robotics. This scale contemplates three dimensions: warmth, competence, and discomfort. This scale is considered to be more valid than NARS in some contexts, since low scores in one of the dimensions do not imply high scores in another, perhaps relevant to the robot’s activity.

Finally, the RAS (Robot Anxiety Scale) [16,17] is another scale that is used to determine the anxiety that occurs in a person when interacting with a robot. It includes various assessment items, e.g. how the robot acts, moves, or speaks to a person during the interaction.

The USUS [18] (Usability, Social acceptance, User experience, and Societal impact) Evaluation Framework is an essential reference in the evaluation of Human-Robot interaction. This framework addresses various evaluation factors: Usability, Social Acceptance, User Experience, and Social Impact. In particular, in the evaluation indicators of Social Acceptance, it includes aspects, such as the Expectation of Performance, Expectation of Effort, Attitude towards Technology, Self-efficacy, Forms of Grouping, Caring, and Reciprocity. In the evaluation modalities, laboratory experimentation with the external observation of an expert and the use of Wizard of Oz Techniques [19] in robots are validated.

The Almere Model [20] is an acceptance test model of technology acceptance of assistive social agents by elderly users that includes variables that relate to social interaction. The Almere Model is based on the Technology Acceptance Model (TAM) [21], which maps the influences on users’ intention to use the technology and the actual use. The Almere Model uses a questionnaire to measure several social aspects (trust, attitude towards technology, perceived sociability, perceived usefulness, among others) that have impact on the predisposition to use a robot. Our proposal aims to externally measure the real inclination to use a robot by exposing the patient to the technology. We think that this will give us better measures of this predisposition.

## 3. Proposal

In this section, we describe an acceptance test of a robot used in therapies for people with dementia. This work aims at proposing guidelines for evaluating the suitability of a care robot in these tasks. The proposed method is not particular to any therapy or robot, rather it applies to any social assistive robot.

### 3.1. Research Questions

The purpose of this acceptance test was to investigate the following main research question in social robots: is there a method in a pilot study to quickly obtain social aspects and users attitudes about a robot in human-robot therapeutical scenarios?

This question raises the following two detailed research questions that can be presented as research hypotheses:

$RH_{1}$ Does a simplified test supported in six aspects and three games has a significant positive impact on developers and social science researchers’ understanding of human-robot interaction?
$RH_{2}$ Do these six aspects have any relation with the Mini-Mental State Examination [22] (MMSE) of the patients?

### 3.2. Acceptance Test

We have not defined acceptance tests for specific therapies. We have gathered the possible interactions that a humanoid robot with similar characteristics to Pepper (aspect, a touch screen, speakers, and lights) can carry out with a patient. These characteristics are the basis for defining treatments that are applied to patients and the development of robot behaviors in the patient’s vicinity. In the following list, we will list operations that we consider to be relevant to be evaluated in the proposed test.

**Figure 3:**
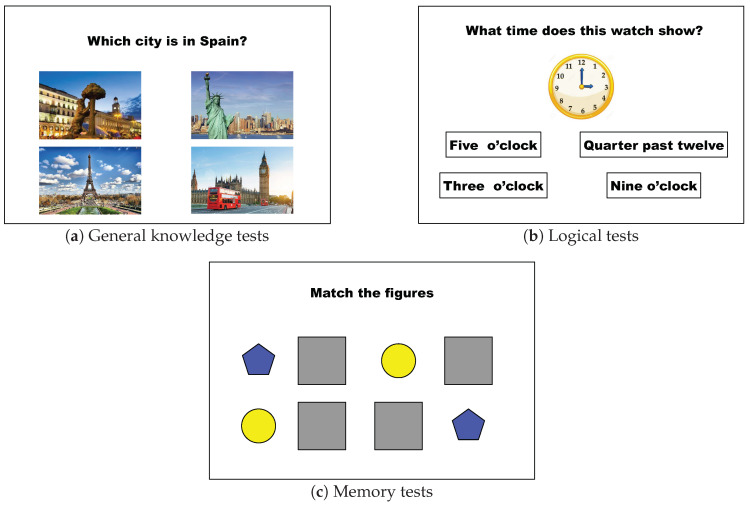
Games that are part of cognitive therapy. (**a**): General knowledge game; (**b**): Logical game; (**c**): Memory game

1. The robot will be present in the patient’s environment, either standing or in motion, navigating from one point to another in the environment.
2. The robot must interact with patients autonomously, or assisted by a therapist.
3. The robot will use its voice to address the patient to give instructions or to ask questions.
4. The robot will be able to perform choreographies that include movements and music.
5. The patient will communicate with the robot mainly through the tablet on its chest.
6. The robot will present games on its tablet with different objectives in which the patient will participate using the tablet in a tactile way. The games’ aim is twofold: on the one hand, to cognitively stimulate the patient. On the other hand, the robot will be able to save the results (success, response time) of each attempt, establish the evolution of the measured variables over time, and determine the degree of acceleration of the patient’s deterioration. Figure 3 shows the interface of the tests displayed in the robot’s tactile screen.

Interacting circumstances have different effects on users. These effects depend strongly on subjective and objective parameters. Extending Ardito’s research [23], the changes to the interacting condition come from four factors:

- environmental factors: where the experiment is performed and the physical characteristics;
- individual factors: what are the individual expectations of the robot;
- software factors: functionalities available and performance; and,
- hardware factors: robot performance, appearance, or shape for interacting.

This research avoids the environmental factors of our acceptance tests. It focuses on those associated with the individual and his/her perception of the robot (software and hardware). To that end, we have selected a set of six subjective elements extracted from previous researches [24]. Besides, we have reviewed current works for measuring usability and acceptance in Human-Robot Interaction, from physical [25] and psychometric [26] perspective. Bechade et al. work [27], which uses Pepper, takes into account user feelings during the interaction and global view on the interaction following. The Technology Acceptance Model (TAM) adaptation that was performed by Koceski et al. [28] is used to determine perceived ease-of-use and perceived usefulness associated with the individual attitude towards the technology. The multi-questionnaire approach presented in [29] defines a user-centered design approach of a multi-modal user interface analyzing different interaction modalities. They evaluate this approach based on three questionnaires. Finally, the Unified Theory of Acceptance and Use of Technology (UTAUT), the System Usability Scale (SUS), and ad-hoc approaches are, in the authors’ opinion, hard to accomplish by individuals with special needs. Of course, we have evaluated alternative methods non-based in questionnaires as the one proposed in CLARC [30] or based on multi-perspective [31].

Six aspects support the subjective metrics applied in this research:

- Contact: this aspect refers to the patient’s predisposition to touch the robot. The objective of evaluating this aspect is because many therapies involve the patient touches the robot. We plan to carry out exercises and games in which the patient reaches parts of the robot, especially the hands, and thus be able to measure the patient’s speed and reaction.
- Static Affinity: this aspect measures whether the robot arouses negative feelings (fear, mistrust) with the robot turned off. This aspect would be the first level of acceptance to measure since a negative score would mean that the application of the robot with that patient would not be advisable.
- Dialogue: this aspect measures whether the person understands the robot’s voice and can assimilate its questions and instructions. A low score in this regard assumes that a therapist would be required to repeat the robot’s explanations or questions. In this test, the robot’s default synthetic voice should be used. This voice is much clearer than in any robot we have used, although it lacks the natural voice’s intonation. One of our future objectives is the generation of a synthetic voice that is more faithful to reality.
- Dynamic Affinity: this aspect refers to the affinity that the robot arouses when it moves. Whether it is waking up, moving their arms, moving around, or doing choreography.
- Perceived Sociability: this generic term refers to the general affinity perception that the robot arouses in the patient if the patient “humanizes” the robot (if (s)he speaks to it as if a person was concerned) and if the patient develops sympathy for it.
- Touch Interaction: this aspect refers to the patient’s ability to interact with the robot through the tablet correctly and effectively. Unreliability of pressing the display and obtaining a response, latency since the screen is pressed, and response are obtained.

The objectives metrics applied in this research are supported in gaming approaches. Particularly, three different games are used:

- Knowledge game: the first game (Figure 3a) is a set of questions with multiple answers, the theme of which explores cognitive abilities related to vocabulary, knowledge of the world, knowledge of the h, and the calendar, and objects common on the daylife.
- Logic game: the second game (Figure 3b) that the robot presents to the patient on its tablet explores the cognitive aspects of the patient related to calculation, spatial reasoning, and logic. This game is presented as a multi-answer trivia game.
- Memory game: this aspect evaluates the patient’s capacity for short-term retention [32]. It is a matching pairs game with four pairs of simple geometric figures (Figure 3c).

**Figure 4:**
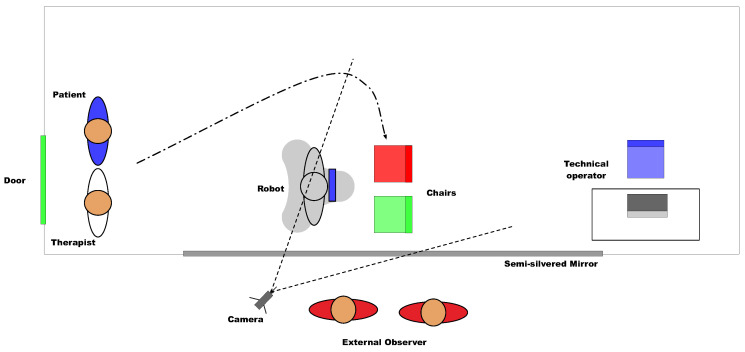
Scheme of the scenario of the experiment.

We have designed an acceptance test to evaluate these aspects, whose scenario is shown in Figure 4. It is a room that is equipped with a one-way mirror that allows vision only towards the test space. On the other side of the glass, there is a camera to record the session and one or more experts, observers. The robot is located in the center of the room. There are two chairs in front of the robot, one for the participant and another for the therapist. The distance to the robot is such that it allows the robot to move without encountering obstacles. Still, it will enable manipulation of the robot’s tablet without making great efforts. Behind the chairs, an operator will control the robot’s movements, generate dialogue with the patient, and control the phases of the games (using the Wizard of Oz technique).

The role of the therapist in carrying out the test is very relevant. In addition to guiding the patient through the process, he should ask at each stage of the test how the patient feels, if (s)he liked the current interaction and if she wants to continue the experiment. External observers take these responses and their observations as the basis for the scores.

#### Instrumentation

The main assessment instrument was a 20 item questionnaire (Table 2) based on Likert-scale questions [33]. This scale has been validated to be used in questionnaires that measure psycho-social aspects. The expert rates the degree of agreement with the statements on a fve-point Likert scale, with one entirely disagree and five fully agree.

## 4. Experiments

This section shows the results of applying the test described above to patients of the State Reference Center for Care for People with Alzheimer’s Disease and other Dementias (CREA) of Imserso in Salamanca on 28th November 2019.

### 4.1. Demography

**Figure 5:**
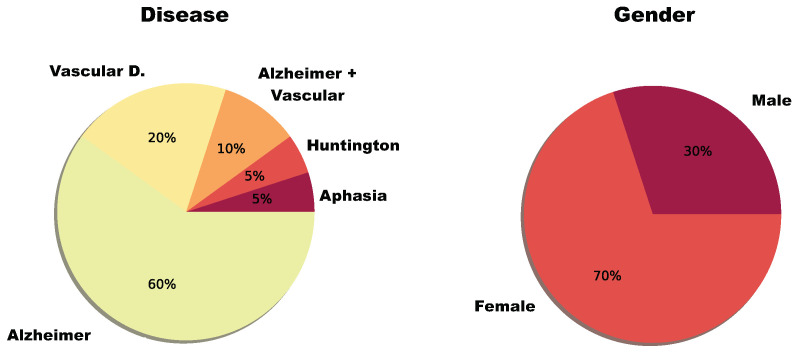
Distribution by diagnosis and by gender of the people who participated in the study. (**left**):population disease distribution; (**right**): gender distribution.

Twenty patients participated in this study. There were 14 women and six men who presented to this study between 39 and 88 years old. They were affected by one or multiple types of dementia: primary progressive aphasia, Alzheimer’s, Huntington’s disease, and Vascular disease (Figure 5).

**Figure 6:**
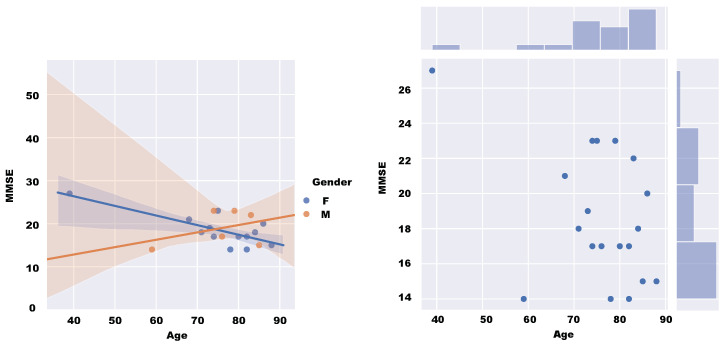
Distribution of subjects by score on the MMSE, and its relationship with age and gender. (**left**):gender distribution; (**right**): age distribution.

Figure 6 shows the characteristics of each subject for the Mini-Mental State Examination (MMSE). According to the Diagnostic and Statistical Manual of Mental Disorders (DSM), this scale is commonly used in diagnosing dementia. This value is calculated from a written test. The maximum score is 30, and low scores indicate severe cognitive problems. The figure shows the relationship between age, gender, and MMSE of the patients subjected to the test.

### 4.2. Robot

We have used a humanoid robot Pepper (Figure 1), This robot is commonly used in social robotics, and is available worldwide at an affordable price given its characteristics ( 15K). It is a 1.20 m high robot, when it is upright, and it weighs 28 kg. Its size is of crucial importance [34], as it is enough to interact with a standup person, and it is not so large to pose a threat. It has a 30.0 Ah/795 Wh battery that allows it to operate for 12 h. It has a 10.1-inch touchscreen on the chest. It has 20 degrees of freedom, and it can move omnidirectionally. The power of the motors in the arms is minimal. It cannot lift weights higher than 100 g, which provides some security in close interaction. The robot runs a Linux operating system and is programmed using the NaoQi framework. It can run both on-board applications and be remotely controlled. It has connectivity by Ethernet cable and wireless.

### 4.3. Description

The experiment relied on a fully experimental approach design supported by a single test. The study protocol was as follows:

1. The patient does not know in advance what (s)he will be facing. (S)He has only been told that it will be a surprise.
2. The robot starts in the position indicated in Figure 4. The technical operator is sitting without interacting with the test participants. The patient and the therapist both enter the room.
3. The patient and the therapist go to the chairs while the therapist dialogues with the patient, directing his/her attention to the robot.
4. Once seated, the therapist invites him/her to touch the robot, asking the patient what (s)he thinks.
5. The robot turns on and performs a sequence of movements that includes movement of the head, arms, and turn on itself.
6. In this phase, for 1–2 min, the therapist encourages the patient to dialogue with the robot. The robot greets and asks questions about the name, where the patient is from, etc. Besides, it responds to the patient’s questions and comments. All of this dialogue is generated by the operator sitting behind the test participants.
7. The robot proposes to play a game, and the Knowledge game, described above, begins. The therapist can help the patient by repeating the questions that (s)he does not understand. However, it is a priority for the patient to select the correct answers autonomously using the robot’s tablet. The robot will use its voice to offer feedback on each question.
8. The robot proposes playing another game. The patient can refuse if his/her experience has not been pleasant with the previous game, skipping the test to the post-game phase. If the patient agrees, the Logic game starts.
9. The robot proposes the last game, which is the Memory game.
10. After the games, the robot asks the patient whether (s)he wants to see how it performs Tai Chi, which is an animation that includes movement, music, and movement of arms. Because Tai Chi is common among the activities of some residences, the therapist can encourage the patient to get up and imitate the robot. After Tai Chi, the robot performs two more animations, increasing the level of movement and noise.
11. The robot says goodbye, and the test participant leaves the room.

**Figure 7:**
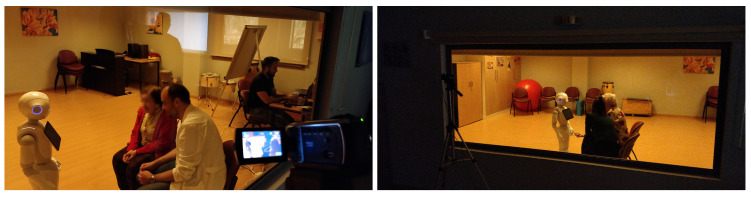
Images during tests.

The total duration of the test is 8–10 min, being observed and recorded from the neighboring room through the one-way mirror (Figure 7).

### 4.4. Results

**Figure 8:**
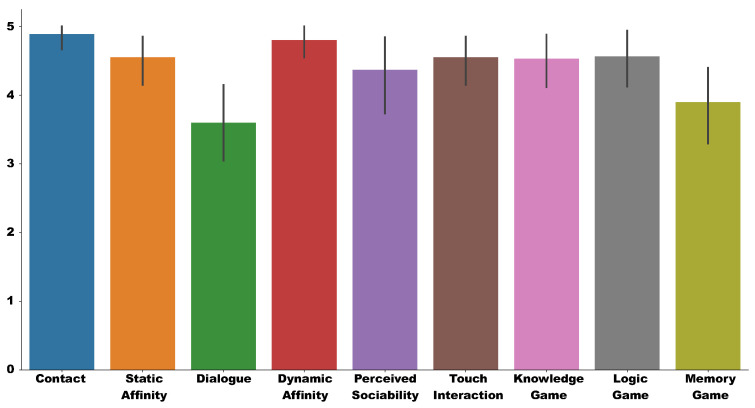
Result of the obtained scored in the test.

Twenty test sessions and the proposed questionnaire were completed during the experimental phase. The scores obtained in the questionnaires were processed, as described above, with values from one to five for each user. Table 3 presents the standard descriptive statistics for obtained scores. Figure 8 shows the mean and standard deviation of the score on the Likert Scale graphically for each of the aspects measured in the test. As shown in Table 3, the mean in most of the characteristics is above 4. The values whose means are lower correspond to the interaction aspects.

**Figure 9:**
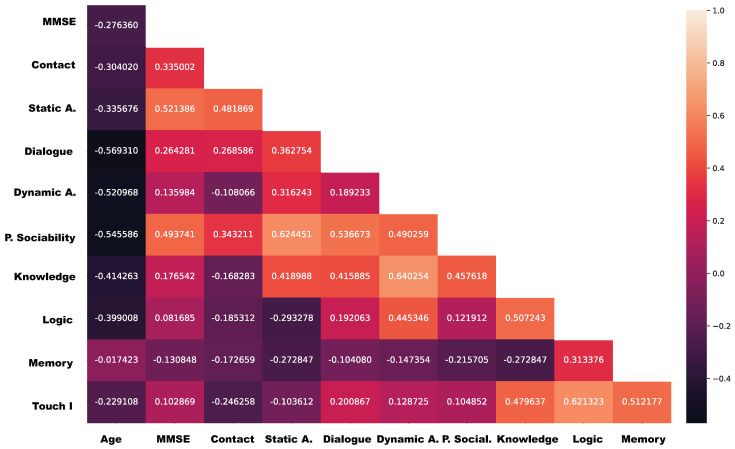
Spearman’s correlation of test results and patient characteristics.

In the case of Dialogue (3.60±1.35), it is observed that voice synthesis fails in patients who have some difficulty in hearing, which is one of the senses affected by dementia. We need to complete the natural voice synthesis module, projected as future works, to generate dialogue dynamically, if we want to avoid pre-recorded voices. The score in the dialogue section is correlated with the score in most of the other sections, except for the Memory game, as shown in Figure 9. If we look at the diagnostic scores (Figure 8), it is observed that only in the case of Huffington’s Disease and Mixed Dementia, the score continues to be high.

In the case of Touch Interaction (3.90±1.33), this score is low. Although patients are used to (although with difficulty) using touch devices in their activities at CREA, these values are not acceptable because the robot’s tablet does not have an optimal response to pulsations. This reason made three patients out of 20 not want to participate in more than one game. The Touch Interaction is also correlated with the rest of the test scores, as shown in Figure 9.

The rest of the test scores are high (greater than 4), with the maximum score being the most common of all the test sections, as shown in Table 3. The item Perceived Sociability (4.36±1.25), which validates the use of the robot in therapies, is remarkable.

Regarding the Contact item (4.88±0.47), patients are very inclined to touch the robot while it is turned off. A similar value (4.55±0.88) is Static Affinity. Most of the patients found the robot pleasant when it is turned off.

The patients felt very comfortable with the robot’s movement, as seen in the item Dynamic Affinity (4.80±0.52). High values are also observed in the performance of the Knowledge game (4.55±0.82), the Logic game (4.53±0.79), and the Memory game (4.56±0.89).

Besides, we have presented the degree of linear association between the proposed evaluation aspects. The idea is to measure whether there is a relationship between two aspects, and if their behavior answers a causal effect [35]. This idea defines the correlation coefficient between aspects, which is based on Spearman’s correlation coefficient. On the one hand, this correlation coefficient measures the degree to which the aspects change together. The correlation gives us three possibilities: a positive correlation, which indicates that, as the value of an aspect increase the value of the other aspect or variable also increases; a negative correlation, which indicates that when one aspect increases the values of the other aspect or variable decreases; and, no-correlation, which implies that there is no reason for thinking that an aspect affects other aspects. On the other hand, this coefficient is a measure of the scatter of the points around this linear trend: the closer the spread of points to a straight line, the higher the value of the correlation coefficient; the greater the spread of points the smaller the correlation coefficient.

**Figure 10:**
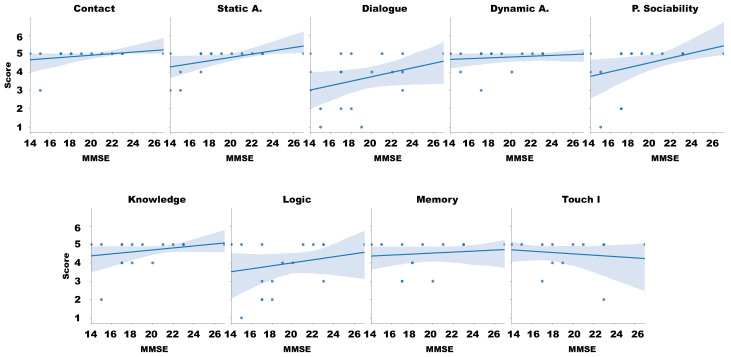
Correlation of Likert test and evaluation factor.

Figure 9 overviews the Spearman’s correlation coefficient between the different aspects analyzed in our questionnaires. Because our questionnaire is independent of the individual needs, we focus our analysis based on the MMSE (Figure 10) and each one of the aspects. This approach provides initial information regarding acceptance and usability of the robot and the experiment and helps the researcher to understand which components should be improved in a secondary iteration.

## 5. Discussion

There are many interpretations that are compatible with our results in the base of the preliminary results of this study. However, these results help us to answer the research hypotheses presented in this study. At first sight, overviewing the scores obtained in the test and illustrated in Figure 8 there are positive results for all aspects under analysis.

We proceeded with a linear correlation attending the scatter-plot between the MMSE and the results of the questionnaire in order to answer the $RH_{2}$. Figure 10 presents that, as the value of MMSE increases, the six aspects of our questionnaire also increase, it means that when the cognitive capacities of the individual are better, also the perception of each aspect is better.

However, the Spearman’s correlation coefficient between the different aspects Figure 9 presents that the MMSE has a weak correlation with Dynamic and Touch aspects, and Knowledge, logic, and Memory games (under 0.2). Subsequently, it has a weak correlation (between 0.2 and 0.4) with contact and dialogue. It has a moderate correlation with static interaction and Perceived Sociability. This leads to accepting that these hesitation in interacting with the robot will increase when those patients with lower MMSE. These results also lead us to think that the approach for performing games with the platform had to be revisited, in particular the Memory Game.

**Figure 11:**
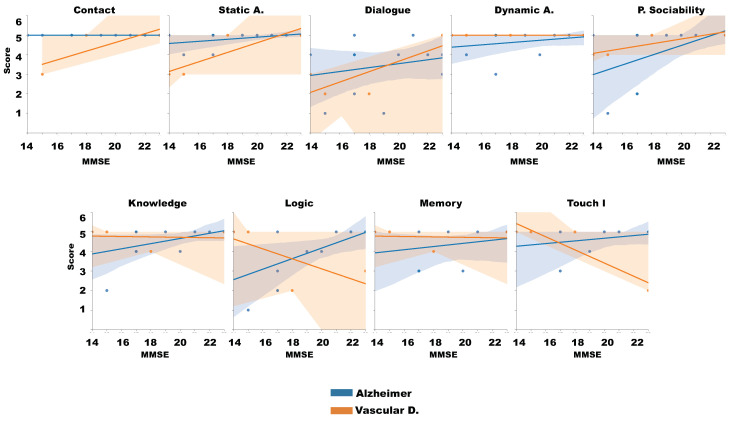
Correlation of Likert test and patient characteristics.

Finally, attending each patient characteristics and focusing on those cases whose population was greater than 2, Alzheimer and Vascular diagnostic, we evaluated the linear relation between the MSSE, the Diagnostic and the questionnaire aspects. Figure 11 illustrates this analysis. In general, those patients with Alzheimer have minimal relation between the aspects and the MMSE, notwithstanding, the vascular individuals have slightly better results with higher MMSE and positive relation with the analyzed aspects, except with the games, which present negative worst acceptation with higher MMSE values.

These results also help us to answer $RH_{1}$, because attending the different MMSE values of our experimental demography, we can remove some of them of our experience if we want to evaluate individual aspects of Human-Robot Interaction (HRI).

### Experts and Patients Opinions

The therapists and psychologists of the CREA of Salamanca highly valued the objectives of this research. They considered that including a social robot in a healthcare environment could make patients’ day-to-day lives more dynamic. They also considered very positive the possibility of recording the results of the games and, thus, being able to assess the evolution of the disease of the patients. They found the acceptance test itself a positive activity for patients.

Therapists and psychologists recommended modifying the robot’s voice to higher pitches in order to be better understood.

As a negative point, therapists and psychologists considered that the robot’s tablet was insufficient for patients to handle it. Although patients often used touch devices, the touch screen did not react appropriately to some interactions. This situation could impact the perception of some games, since it aroused frustration in some cases. Without a doubt, it is a vital element to consider in the future.

## 6. Conclusions

In this document, we present a methodology to carry out robot acceptance tests applied in therapies with dementia patients. This test provides an alternative to tests that are based on direct patient questionnaires, which are sometimes unfeasible due to the patient’s condition. In our approach, acceptance is determined based on observation of the patient’s reaction when interacting with the robot, always guided by a therapist. In this work, we have described the setup of the test, the phases, as well as the sequence of activities that the robot must carry out with the patient. We have provided an observer questionnaire as well as some observation guidelines. The use of the Likert scale contributes to a valid observation methodology.

We have applied this test to a group of twenty patients of different types of dementia in a specialized residential center in order to demonstrate our approach’s validity. The test has been carried out successfully, validating a Pepper humanoid robot for use in subsequent phases of a healthcare robotics project.

The results show that this test is feasible in this type of evaluation, avoiding questionnaires for patients who find it challenging to carry out.

## Figures and Tables

**Table 1:** Negative Attitudes towards Robots Scale (NARS) Questionnaire.

	Question
1	I feel anxiety if robots really have their own emotions.
2	I surmise that something negative for humans happen when robots become more similar to humans.
3	I will be able to be relaxed if I interact with robots.
4	I feel anxiety when I imagine that I may be employed and assigned to a workplace where robots should be used.
5	I will be familiar with robots if they have their own emotions.
6	I am mentally healed when I see robots behaving affectively.
7	I am left helpless even by hearing something on robots.
8	I am likely to bring shame on myself when I use robots in public.
9	The words “artificial intelligence” or “decision by robots” make me feel unpleasant.
10	Even standing in front of robots will strain me.
11	I surmise that extreme dependence on robots may cause something negative for humans in future.
12	I will feel nervous if I interact with robots.
13	I am afraid that robots may negatively influence children’s mind.
14	I surmise that future societies may be dominated by robots.

**Table 2:** Questions of the questionnaire and its relation with the evaluated aspects

Question		Aspect Evaluated
1	Does the patient show fear in touching the robot when it is turned off?	Contact
2	Does the patient show fear in touching the robot when the robot begins to move?	
3	Does the patient show any hesitation in interacting with the switched off robot?	Static Affinity
4	Does the patient show any qualms about sitting near the switched off robot?	
5	Does the patient understand the spoken instructions of the robot?	Dialogue
6	Does the patient respond directly to the robot?	
7	Does the patient perform a fluid interaction, without need for assistance?	
8	Is the patient scared or restless when the robot begins to move?	Dynamic Affinity
9	Does the patient find the robot’s choreography pleasant or funny?	
10	Does the patient show a good predisposition towards the robot initially?	Perceived Sociability
11	Is the patient comfortable with the robot during the session?	
12	Does the patient want to interact with the robot again in the future?	
13	Does the patient interact fluently with the robot through the touch tablet?	Touch Interaction
14	Does the patient require assistance to use the robot’s tablet?	
15	Does the patient understand the dynamics of the game without assistance?	Knowledge Game
16	Does the patient want to play another game at the end of knowledge game?	
17	Does the patient understand the dynamics of the game without assistance?	Logic Game
18	Does the patient want to play another game at the end of the logic game?	
19	Does the patient understand the dynamics of the game without assistance?	Memory Game
20	Does the patient want to play another game at the end of the memory game?	

**Table 3:** Statistical results of the tests.

	Mean	Standard deviation	Median	Mode	Min	Max
**Age**	75.40	11.03	77.0	74	39	88
**MMSE**	18.63	3.71	18.0	17	14	27
**Contact**	4.88	0.47	5.0	5	3	5
**Static affinity**	4.55	0.88	5.0	5	2	5
**Dialog**	3.60	1.35	4.0	4	1	5
**Dynamic affinity**	4.80	0.52	5.0	5	3	5
**Perceived Sociability**	4.36	1.25	5	5	1	5
**Physical interaction**	3.90	1.33	4.5	5	1	5
**Knowledge Game**	4.55	0.82	5.0	5	2	5
**Logic Game**	4.53	0.79	5.0	5	3	5
**Memory Game**	4.56	0.89	5.0	5	2	5
